# Foreign Body Reaction to Neural Implants: A Comparative Study of Polymer Toxicity and Tissue Response

**DOI:** 10.3390/bios15090599

**Published:** 2025-09-11

**Authors:** Ciara Makievskaya, Anna Brezgunova, Nadezda Andrianova, Evgeny Kelm, Maria Solovyova, Gelena Naumova, Alina Zeinalova, Olga Gancharova, Tatiana Bushkova, Daniil Kozlov, Valery Putlayev, Pavel Evdokimov, Alexander Petrov, Mikhail Lebedev, Egor Plotnikov, Vasily Popkov

**Affiliations:** 1 Faculty of Bioengineering and Bioinformatics, Lomonosov Moscow State University, 119192 Moscow, Russia; c.makievskaya@iai.msu.ru; 2 Institute for Artificial Intelligence, Lomonosov Moscow State University, 119992 Moscow, Russia; brezgunova@belozersky.msu.ru (A.B.); andrianova@belozersky.msu.ru (N.A.); kelm87@yandex.ru (E.K.); mashike33@yandex.ru (M.S.); naumova.gelena@gmail.com (G.N.); petrovak@my.msu.ru (A.P.); plotnikov@belozersky.msu.ru (E.P.); 3 A.N. Belozersky Institute of Physico-Chemical Biology, Lomonosov Moscow State University, 119234 Moscow, Russia; ogancharova@cspfmba.ru; 4 Federal State Budgetary Institution “Centre for Strategic Planning and Management of Biomedical Health Risks”, Federal Medical Biological Agency, 119121 Moscow, Russia; azejnalova@cspfmba.ru; 5 Chemistry Department, Lomonosov Moscow State University, 119234 Moscow, Russia; tania.bushkova@yandex.ru (T.B.); valery.putlayev@gmail.com (V.P.); pavel.evdokimov@gmail.com (P.E.); 6 Kurnakov Institute of General and Inorganic Chemistry of the Russian Academy of Sciences, 119991 Moscow, Russia; kozlov@inorg.chem.msu.ru; 7 Materials Science Department, Lomonosov Moscow State University, 119192 Moscow, Russia; 8 Faculty of Physics, Lomonosov Moscow State University, 119234 Moscow, Russia; 9 Faculty of Mechanics and Mathematics, Lomonosov Moscow State University, 119234 Moscow, Russia; mikhail.a.lebedev@gmail.com

**Keywords:** toxicity, polymers, neural interfaces, neurons, foreign body reaction, chronic implantation

## Abstract

This study investigated the toxicity of ten polymer materials intended for the development of invasive neural interfaces improving the treatment of neurological diseases. Most of the materials for neural implants can cause traumatization of the surrounding tissue, inflammation, and foreign body reaction. In this study, in vitro and in vivo toxicity assessment was performed for nylon 618 (NY), polycaprolactone (PCL), polyethylene glycol diacrylate (PEGDA), polydimethylsiloxane (PDMS), polyethylene terephthalate (PET), polylactide (PLA), thermoplastic polyurethane (TPU), polypropylene (PP), polyethylene terephthalate glycol (PET-G), and polyimide (PI). The biocompatibility of these ten materials was assessed based on cell adhesion, growth and cytotoxicity on neural (PC-12) and fibroblast (NRK-49F) cultures. Furthermore, brain tissue responses to the implanted phantom scaffolds were analyzed in rats. According to these measurements, PI showed the highest compatibility for both cell types. PEGDA exhibited cytotoxic effects, low cell adhesion and the strongest foreign body reaction, including fibrosis and multinucleated cell formation. The other polymers showed lower pathological responses which makes them potentially usable for neural interfacing. We conclude that PEGDA appears to be unsuitable for long-term use due to adverse tissue and cellular reactions, whereas PI, PLA, PDMS and TPU hold promise as materials for safe and effective neural interface applications.

## 1. Introduction

Invasive neural interface technologies have been actively developed as an approach to the treatment of numerous diseases of the central and peripheral nervous system [1]. Their potential for medical applications, such as restoring impaired sensory or motor functions, as well as fundamental neurobiological research, has increasingly gained recognition [2]. Invasive neurotechnologies are applicable to the treatment of a number of neurological pathologies, in particular, chronic pain, epilepsy, paralysis, depression, Parkinson’s disease, and sensory disabilities, such as loss of hearing and vision [3].

To sample electrical brain activity, invasive neural interfaces require conductive elements in combination with the polymer substrates that form the mechanical framework of the device and protect the electrically conductive components [4]. Because such polymers interact directly with the neural tissue, the biocompatibility of the devices crucially depends on the properties of these materials [5]. For a device to be effective in the long term, it must have surface and structural biocompatibility and be biostable over a long period of time [6].

The biocompatibility of a material is a complex characteristic that depends on both its toxicological safety for the host and the degree of mechanical compatibility between the material and surrounding tissues. One of the main mechanical properties of the material in the context of biocompatibility is stiffness, expressed in Young’s modulus. While Young’s modulus of brain tissue is close to 1.0 kPa [7], the most common electrodes are made of metals and silicon, which have a Young’s modulus of around 100–200 GPa [8]. Polymeric and hydrogel-based electrodes are promising due to their significantly lower rigidity.

In this study, we focus specifically on assessing the toxicological safety of materials. We assessed the toxicity of different materials to nearby cells, morphological changes when a large foreign body was introduced into the nervous tissue, and acute and chronic reactions to that foreign body [9]. The foreign body reaction (FBR), which includes a complexity of events, is the major limiting factor for the practical implementation of neural interfaces. New materials are needed to solve the problem of biocompatibility and widen the implementation of invasive interfaces in clinical practice [10]. A number of polymer-based materials have been proposed as the insulating layer for neural interfaces. Some of them are already being used in clinics, others are being tested in animal models, and still others are just promising laboratory samples that need to be tested further.

The biocompatibility of polymers in neural interfaces is conventionally examined with two groups of tests: in vitro experiments on cell cultures, and in vivo experiments on animals [11]. In the cell-culture tests, the main goal is to assess material toxicity to the living cells, primarily neural cells, and investigate the physical interaction of the cells with the surface of the surface polymer structure. Animal experiments are used to identify more complex mechanisms of interaction between the polymers and the organism at the local and systemic levels. A comprehensive analysis of the FBR in vivo involves the determination of immune responses, primarily the inflammatory response, the adhesion of various proteins to the polymer surface, and the formation of a fibrous capsule or gliomesodermal scar around the embedded structure [12].

The main limitation of the previous biocompatibility studies was that they tested no more than 1–2 polymers simultaneously, so the comparison of many polymers could be made only based on the results of different groups obtained from different experimental models. In the present study, we overcame this limitation by testing 10 polymers simultaneously under the same conditions in both in vitro and in vivo samples. We examined several previously tested materials together with the previously unexplored materials with Young’s modulus within hundreds of MPa, which could be implemented in 3D-printed interfaces. Among the ways to manufacture neural interfaces, 3D printing is especially efficient because with this method many different polymers can be handled with the same technological process, including building implants composed of several different materials. Accordingly, we used 3D printing to produce the experimental samples of scaffolds.

The aim of this work was to evaluate and compare cellular and tissue responses to ten polymers within one experimental framework. We compared the effects of scaffolds on the growth and proliferation of two cell types placed on their surface: neural cells and fibroblasts. For the same samples, we examined the surface of the scaffolds using SEM. We also assessed the possibility of cytotoxic compounds being released by the substrates into the cell culture. In the in vivo experiments, we used brain implants made of these materials to examine their biocompatibility and FBR after four post-implantation weeks.

## 2. Results

### 2.1. Surface Properties of the Scaffolds

**Figure 1:**
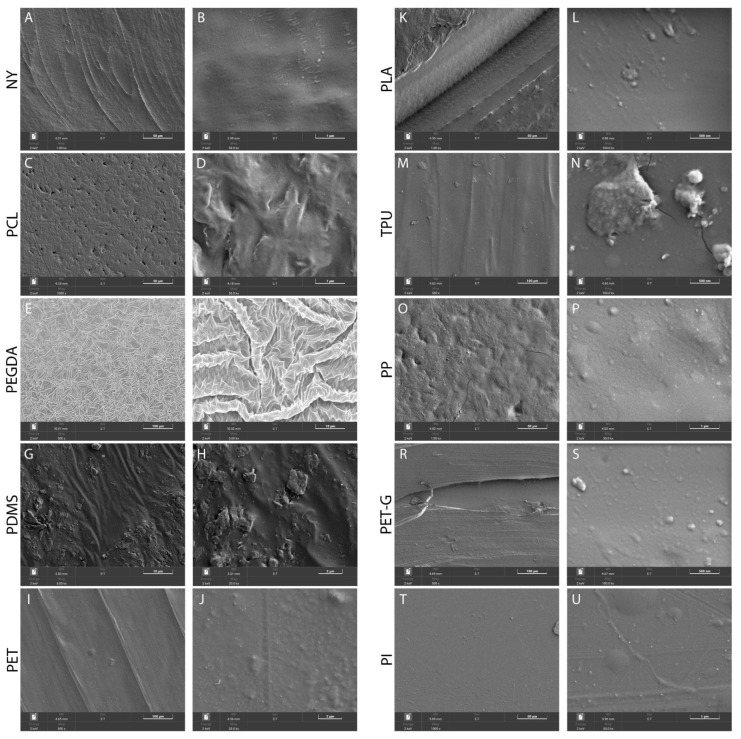
The assessment of the surface structure of the scaffolds composed of (**A**,**B**) NY, (**C**,**D**) PCL, (**E**,**F**) PEGDA, (**G**,**H**) PDMS, (**I**,**J**) PET, (**K**,**L**) PLA, (**M**,**N**) TPU, (**O**,**P**) PP, (**R**,**S**) PET-G, and (**T**,**U**) PI. SEM images of the substrate surface are shown at high and low magnification. Scale bars—50 μm (**A**,**C**,**K**,**O**,**T**), 1 μm (**B**,**D**,**P**,**U**), 100 μm (**E**,**I**,**M**,**R**), 2 μm (**H**,**J**), 10 μm (**F**,**G**), and 500 nm (**L**,**N**,**S**).

The SEM revealed individual extended bulges and pores elongated in one direction on the surface of the NY sample (Figure 1A). The strands protruding on the surface were probably the surface of the material fused during thermal extrusion, and therefore, their directions corresponded to the direction of the build-up of the upper layer formed during the sample printing.

Separately, large pores of about 20–200 microns in size could be observed extending along the direction of the fibers. The formation of such pores was probably caused by the ruptures in the molten polymer after thermal extrusion during the attachment to the previous layer. At high magnification (Figure 1B), individual polymer fibers with a diameter of about 50 nm could be observed, which consisted of larger aggregates extending along the build-up direction of the upper layer. There was also a number of small particles 10–100 nm in size on the surface, which probably represented individual polymer aggregates.

In contrast to most samples obtained by thermal extrusion, the morphology of PCL was isotropic, so it was impossible to draw a conclusion about the direction of the upper-layer structure. At low magnifications (Figure 1C), individual pits were seen on the surface of the sample, and the surface itself was wavy. This morphology could be due to the shrinkage of the polymer during cooling after the thermal extrusion. The presence of scratches on the surface of the sample was not related to its production by three-dimensional printing; rather, it was a defect formed during storage. At high magnification (Figure 1D), individual pits and a wavy surface structure were seen. At the same time, there were no obvious aggregates. Isotropic particles, with a size of less than 30 nm, were present on the surface, which were probably individual polymer beads. In the areas where the surface layer was destroyed (Figure 1D), the layered arrangement of individual flocculant polymer particles was seen.

The surface of the PEGDA sample was a folded structure (Figure 1E) that formed during the drying of the hydrogel sample. At high magnifications (Figure 1F), the folded structure was also present. During the subsequent swelling, the polymer chains straightened, which led to the smoothing of the hydrogel surface.

On the surface of the PDMS sample, at low magnification (Figure 1G), both separate smooth areas with wavy morphology and faceted aggregates up to 5 μm in size were observed. The overall wavy morphology of the surface had no clear direction due to the shrinkage of the sample during cooling after pouring into the mold. At high magnifications (Figure 1H), separate aggregates consisting of isotropic particles with a size of about 20–200 nm were present. It is worth noting that some of the aggregates were partially hidden under the surface of the polymer, so it can be assumed that they were present throughout the volume of the resulting sample.

In the SEM images of the PET sample, at low magnifications (Figure 1I), individual beads were seen on the surface of the sample which were elongated in one direction. The protruding strands on the surface were probably the boundary of the material fused during thermal extrusion, and therefore, their directions corresponded to the direction of build-up of the upper layer during printing of the sample. There was no pore formation on the surface of the sample, which meant that the attachment of the polymer melted after thermal extrusion occurred without the formation of defects. At high magnification (Figure 1J), individual polymer fibers with a diameter of about 50–150 nm were observed, consisting of larger areas elongated along the build-up direction of the upper layer. There were also areas on the surface where the formation of flat isotropic regions with lateral dimensions of 0.5–2 μm were observed. These regions could have been caused either by mechanical spalling due to suboptimal thermal conditions (local overheating) during the 3D printing or by oxidation of the hot polymer surface.

The PLA images clearly showed that the extruded polymer was not effectively attached to the previous layer during three-dimensional printing, resulting in significant cavities. At low magnifications (Figure 1K), considerable destruction of the sample surface was seen near the boundaries of the fusion areas. Individual polymer fibers were clearly visible at the edges, while the surface away from the edges was predominantly smooth. At high magnifications away from the fusion boundary (Figure 1L), the polymer fibers could no longer be distinguished, and individual small particles of about 20–200 nm in size were observed, which were presumably separate polymer aggregates.

The TPU sample, like other thermo-extrusion polymers, had directionally bound convex regions that represented the boundary of the material fused during thermal extrusion and whose directions therefore corresponded to the build-up direction of the upper layer when the sample was printed (Figure 1M). There was no pore formation on the surface of the sample, which means that the attachment of the polymer melted after thermal extrusion had taken place without the formation of defects. At high magnifications (Figure 1N), cracks and a small number of aggregates were seen on the surface of the sample. The latter consisted of isotropic particles with a size of 50–200 nm.

The morphology for PP was similar to that for PLC. It was isotropic, and it was not possible to draw a conclusion about the build-up direction of the upper layer. At low magnifications (Figure 1O), pits, separate smooth areas, and isotropic convex areas of approx. 1–5 microns in size were seen on the surface of the sample. At high magnifications (Figure 1P), small particles with a size of about 10–100 nm were observed in addition to the convex areas.

The SEM images of the PET-G sample showed a similar pattern to PET. Individual flat, elongated bulges could be seen on the surface of the sample, which extend in one direction (Figure 1R). The elongated areas protruding on the surface were probably the surface of the material that was melted during thermal extrusion, and therefore, their directions corresponded to the build-up direction of the top layer when the sample was printed. Large pores were observed separately. They flew into the cracks that were about 200 microns in size and extended along the direction of the fibers. The formation of such cracks was probably caused by the ruptures in the molten polymer after the thermal extrusion during the attachment to the previous layer. At high magnifications (Figure 1S), individual polymer fibers with a diameter of about 50 nm were observed, consisting of larger areas elongated along the build-up direction of the upper layer. There was also a small number of small particles of about 10–200 nm in size on the surface, which were probably individual polymer aggregates.

The PI sample showed predominantly smooth areas and separate isotropic convex regions of about 1–5 microns in size (Figure 1T). The scratches observed on the surface probably formed during storage of the sample and were not related to the process of its production. At high magnifications (Figure 1U), small particles of about 10–100 nm in size were observed in addition to the convex regions.

### 2.2. The Growth of Neuronal PC-12 Cells on the Scaffolds

**Figure 2:**
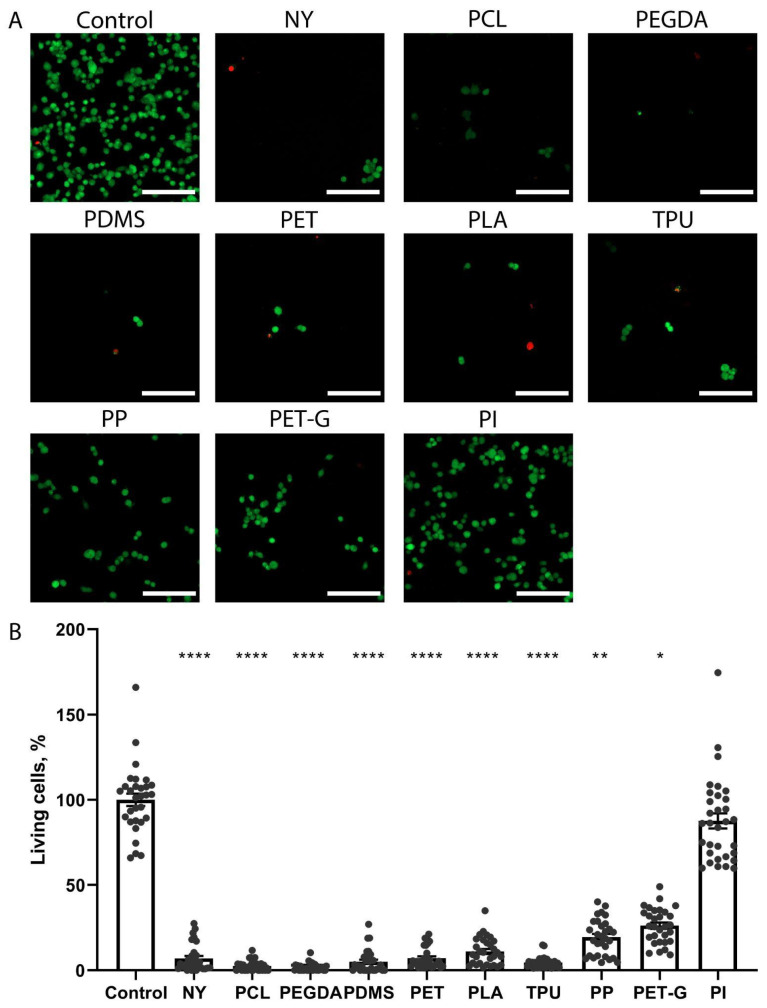
Assessment of adhesion and morphology of PC-12 cells growing in the control well of the tablet or on scaffolds made of polymers NY, PCL, PEGDA, PDMS, PET, PLA, TPU, PP, PET-G, and PI. (**A**). Confocal images of cells stained with calcein (green; reveals living cells) and propidium iodide (red; detects dead cells). Scale bar 100 µm. (**B**). Counting the number of living cells on various substrates according to confocal microscopy data. **** *p* < 0.0001, ** *p* < 0.01, * *p* < 0.05. The groups were compared using the Kruskal–Wallis criterion with multiple Dunn comparisons. The data is presented as an average value ± the standard error of the mean.

Analysis of the growth of the neuronal PC-12 culture on substrates of different materials revealed very different degrees of adhesion and growth of cultures for different materials. Representative confocal images of PC-12 cells stained with calcein and propidium are shown in Figure 2A, and the quantitative image analysis is shown in Figure 2B. The neuron-like cells of the PC-12 culture in the control were positive when stained with calcein and had a compact cell body shape characteristic of this culture. A few dead cells stained with propidium iodide were observed. Single live cells and a similar number of propidium-positive dead cells were present on NY, TPU, PET, PLA, PCL, PDMS, and PEGDA. A total of 5–10 live cells were present in the field of view on TPU. On PET, a small number of live cells stained with calcein were visualized, and single dead cells were present in part of the field of view. On the substrates of PP and PET-G, a certain number of live, attached PC-12 cells were observed, but their number was less than half of the control (Figure 2), and there were also a few dead cells. The neurons grew in the largest numbers on polyimide, and their number and morphology completely matched those of the control samples.

### 2.3. The Growth of Fibroblast NRK-49F Cells on the Scaffolds

**Figure 3:**
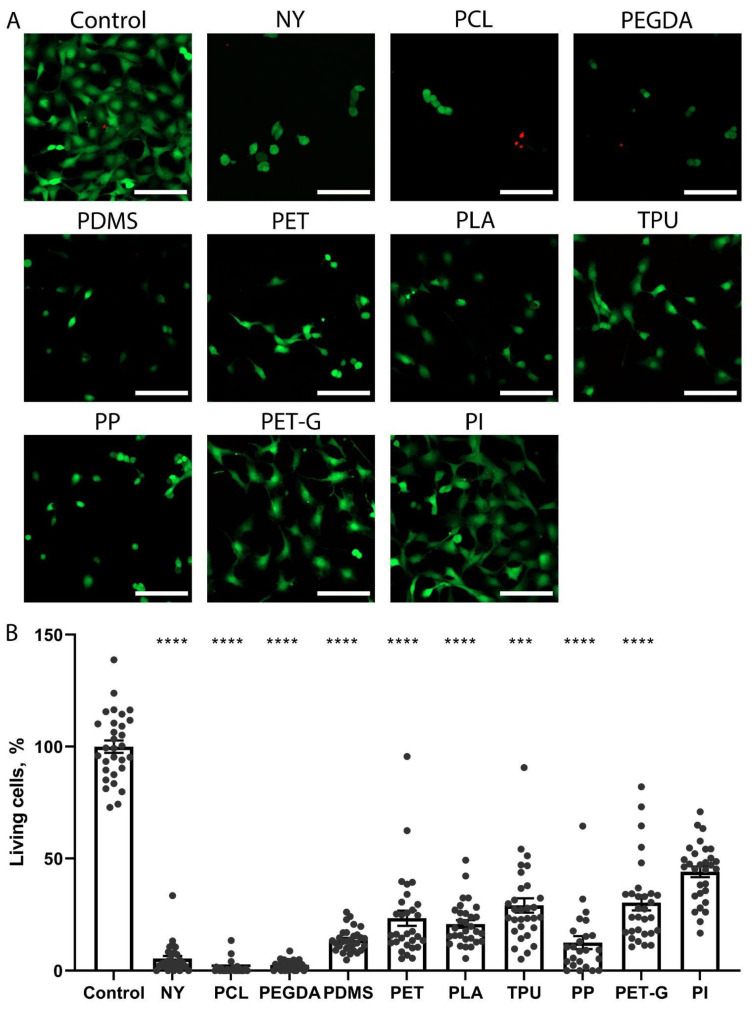
Assessment of adhesion and morphology of NRK-49F cells growing in the control well of the tablet or on scaffolds made of polymers NY, PCL, PEGDA, PDMS, PET, PLA, TPU, PP, PET-G, and PI. (**A**). Confocal images of cells stained with calcein (green; reveals living cells) and propidium iodide (red; detects dead cells). Scale bar 100 µm. (**B**). Counting the number of living cells on various substrates according to confocal microscopy data. **** *p* < 0.0001, *** *p* < 0.001. The groups were compared using the Kruskal–Wallis criterion with multiple Dunn comparisons. The data is presented as an average value ± the standard error of the mean.

The analysis of growth of NRK-49F fibroblasts on substrates made of different materials showed a different pattern (Figure 3) compared to the growth of PC-12 cells. When grown in a standard dish, NRK-49F culture cells had a normal morphology characteristic of the NRK-49F cell line and formed a monolayer. Single propidium-positive dead cells were present. On the NY substrate, the cells practically did not grow, with only very few living cells present in the field of view. There were also fields of view in which numerous dead cells could be seen. A relatively large number of living cells were found for TPU, PET, and PLA. Although the density of the monolayer was not as high as the values for the control, the cells looked well attached and had a characteristic spreading morphology. On all three polymers, single dead cells were present in some fields of view, and their number was comparable to the control. On PP and PDMS substrates, a small number of live, adherent cells were found, some of which had a shriveled, rounded shape, indicating that the cells did not adhere and spread effectively on this polymer. The number of propidium-positive cells was also low. A large number of live cells with a characteristic flattened morphology were observed for the PET-G. For PCL, a very low number of live, adherent cells were observed, all with a shriveled morphology, and dead cells were frequently observed. The pattern observed for PI was the closest to the control. Here, the highest number of live cells among all substrates and single dead cells were observed in some fields of view (Figure 3B). On the PEGDA polymer, there were virtually no cells, and only dead cells were detected.

### 2.4. Comparison of the Toxicity of Substrate Materials

**Figure 4:**
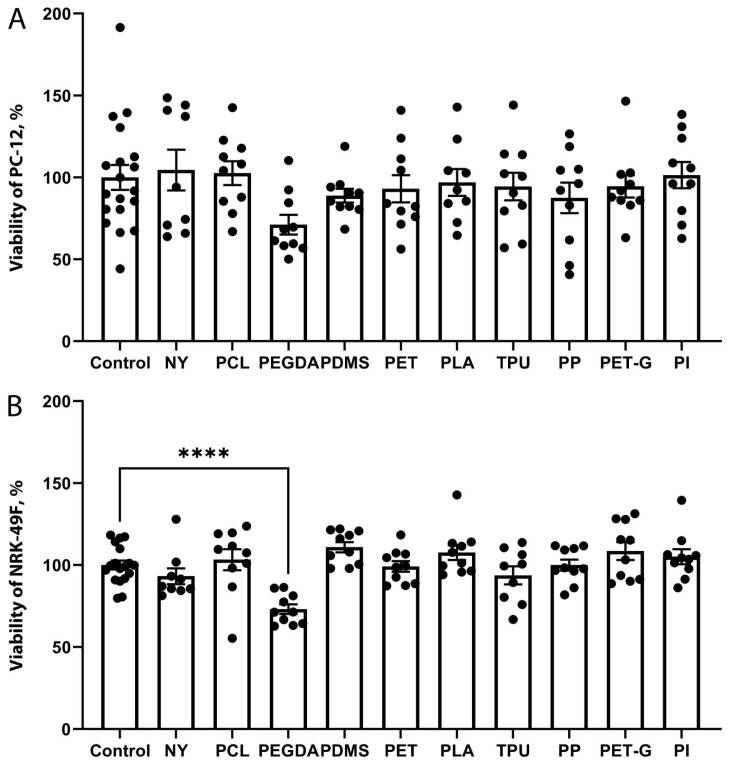
Study of the viability of PC-12 (**A**) and NRK-49F (**B**) cells after incubation with extracts of NY, PCL, PEGDA, PDMS, PET, PLA, TPU, PP, PET-G, and PI polymers for 48 h. **** *p* < 0.0001. The comparison of the groups was carried out using single-factor analysis of variance with multiple Tukey comparisons. The data is presented as an average value ± the standard error of the mean.

To determine whether the lack of cell growth was due to the adhesive properties of the polymer or to the potential toxicity of the material, we performed a viability test on the cells grown on the medium conditioned with selected polymers. As in the case of cell growth on the substrates, PC-12 and NRK-49F cells were incubated for 48 h in a climate-controlled environment. The analysis revealed that most extracts did not affect the viability of the cultured cells (Figure 4). The only polymer that significantly reduced cell viability was PEGDA.

### 2.5. Morphological Changes in the Brain During Implant Placement

**Figure 5:**
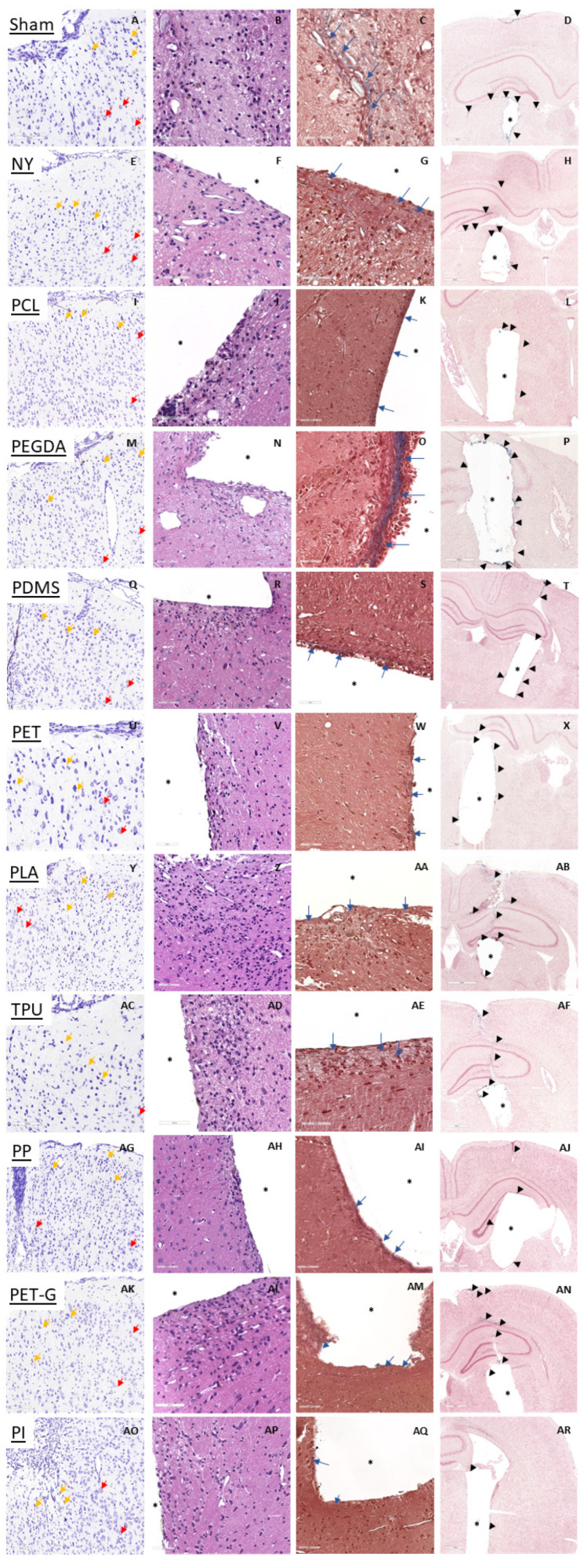
Morphological changes in brain tissue during the implantation of the phantom polymer samples four weeks post-surgery. (**A**,**E**,**I**,**M**,**Q**,**U**,**Y**,**AC**,**AG**,**AK**,**AO**)—Nissl’s staining showing degeneration of neurons at the site of cortex displacement (yellow arrows highlighting degenerated neurons, red arrows highlighting normal neurons, and magnification 200×). (**B**,**F**,**J**,**N**,**R**,**V**,**Z**,**AD**,**AH**,**AL**,**AP**)—Overview of brain tissue morphology near the implant, hematoxylin and eosin staining, and magnification 400×. (**C**,**G**,**K**,**O**,**S**,**W**,**AA**,**AE**,**AI**,**AM**,**AQ**)—Early formation of fibrotic scar, collagen fibers are stained in blue (Masson’s trichrome staining, blue arrows highlighting collagen layer, and magnification 400×). (**D**,**H**,**L**,**P**,**T**,**X**,**AB**,**AF**,**AJ**,**AN**,**AR**)—Perls’ staining, dying hemosiderin in blue, indicating the traces of hemorrhage occurred at the acute phase along the whole implantation channel (black triangles highlight areas containing hemosiderin, and magnification: (**L**)—40×, (**T**,**AN**)—20×, (**other**)—30×). Asterisk corresponds to the cavity developed by the implant.

A separate evaluation was conducted for the effects of surgical intervention needed for the implant placement. The surgery itself caused tissue degeneration and interfered with the FBR. These effects were primarily caused by the displacement and tearing of neuronal tissue components, rupture of blood vessels, and axons [13,14]. Thus, in all groups, including sham-operated animals, degenerated neurons with pyknotic appearance were found alongside the injection channel in the cortex, hippocampus, thalamus, and hypothalamus four weeks post-surgery (Figure 5A,E). Degenerated neurons were abundant near the areas of mechanical damage, but their number rapidly decreased outside of these areas. Following the damage to neuronal tissue, microglia became activated along the damage border. Comparable glial activation was observed in all samples (Figure 5B,F). Notably, the activated glia was not aligned uniformly around the implant, but was gathered into small, rare focuses in the peri-implant area. At the same time, sparse collagen fibers indicated formation of fibrous scar (Figure 5C,G). Collagen fibers did not form a consistent layer along the perimeter of the implant, indicating the mildness of FBR. Finally, as a sign of hemorrhage resolution, many hemosiderophages (macrophages that phagocytized erythrocytes earlier during intraoperative bleeding) were present along the wound channel and hippocampus subfields (Figure 5D,H).

**Figure 6:**
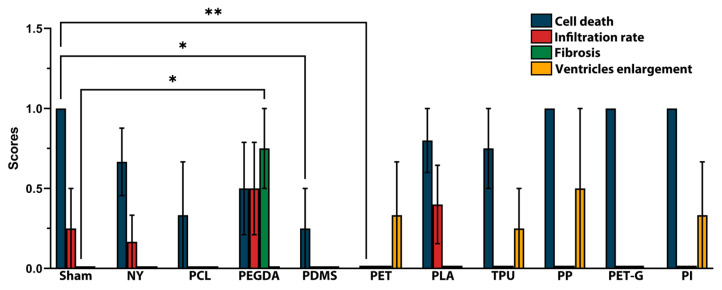
Summary of histological scores obtained 4 weeks after implantation. ** *p* < 0.01, * *p* < 0.05 two-way ANOVA with Dunnett’s multiple comparisons test.

Notably, the quantitative analysis of the histological changes did not reveal any dramatic excess of negative manifestations compared to the sham-operated animals. However, the most pronounced pathological process was cell death, which was observed in the implantation of almost all polymer materials, as well as mild infiltration. Fibrosis was only observed in the case of PEGDA (0.75 ± 0.5 vs. 0 ± 0). Most of the detected alterations were also observed in the sham-operated group, apart from ventricles dilatation. PDMS and PET (0.25 ± 0.5 vs. 1 ± 0 and 0 ± 0 vs. 1 ± 0, respectively) caused significantly lower manifestation of neuronal death compared to sham (Figure 6).

In addition, some polymers caused a slight decrease in pathological signs compared to sham-operated animals. PLA (0.8 ± 0.45) and TPU (0.75 ± 0.5) tend to exhibit milder damage to neural cells compared to sham group (1 ± 0). PCL (0 ± 0), PDMS (0 ± 0), PET (0 ± 0), TPU (0 ± 0), PP (0 ± 0), PET-G (0 ± 0), and PI (0 ± 0) demonstrated no infiltration after 4 post-implantation weeks compared to sham group (0.25 ± 0.5). Meanwhile, PEGDA and PLA showed an increase in infiltrate (0.5 ± 0.58 vs. 0.25 ± 0.5 and 0.4 ± 0.55 vs. 0.25 ± 0.5, respectively).

**Figure 7:**
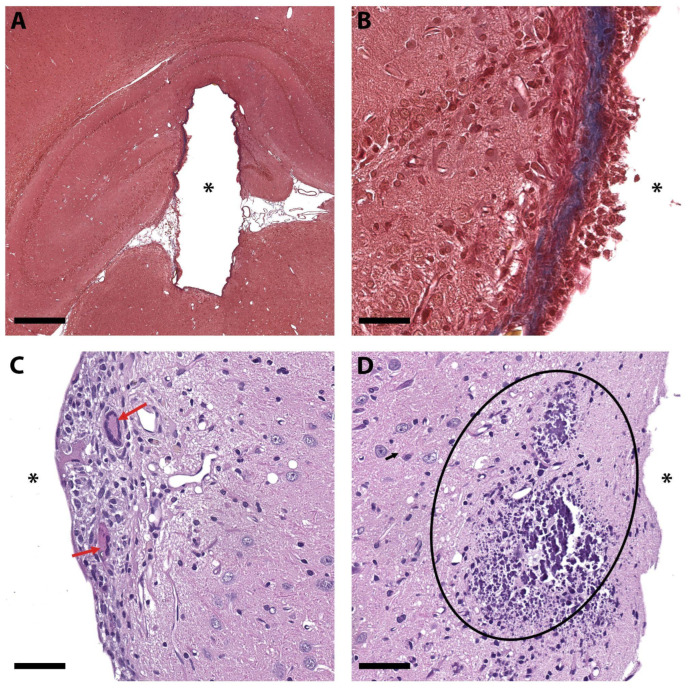
Representative images of the alterations found in the brains implanted with PEGDA. (**A**,**B**)—Formation of fibrotic scar. Collagen is stained in blue; the asterisk marks the implantation site. (**C**)—Inflammatory (granulomatous) reaction to implant, red arrow points to giant multinucleated cell. (**D**)—Migration of implant particles (basophilic particles of different sizes, outlined) up to a certain distance from the implantation site without causing a pronounced inflammatory reaction. (**A**,**B**)—Mallory’s trichrome staining. (**C**,**D**)—Hematoxylin and eosin staining. The asterisk marks the implantation site. Scale bar—500 μm (**A**), 50 μm (**B**–**D**).

The findings were peculiar for the PEGDA group. Only this group showed giant multinucleated cells and macrophages present in the peri-implantation site; the fibrotic capsule covered the entire perimeter of the implant and had a thickness up to 30 µm. In one sample, a small portion of material migrated into the brain tissue near the implantation site, without active inflammation around this area (Figure 7). Altogether, these findings indicate that PEGDA implants caused more prominent tissue reactions compared to the other polymers and, therefore, had the worst prognosis for long-term trials.

## 3. Discussion

Neural interfaces hold promise as an approach to the treatment of a range of neurological diseases [15]. The key requirement for such medical neural implants is that they enable stable performance over long periods of time [16]. Biocompatibility of the implant is critical for this. Biocompatibility can be improved by developing implants that closely match the mechanical properties of neural tissue, especially stiffness, expressed in Young’s modulus [17,18].

Polymers are appropriate for use in brain implants because they could provide long-term stability, low material stiffness (i.e., high material flexibility), and can be both a good electrical conductor or an insulator of the implant [19,20]. The electrode coating has the greatest impact on biocompatibility due to direct contact with the neural tissue. In this study, we analyzed the biocompatibility of 10 polymers used for electrode coating. The experiments were performed in both cell cultures (including cultures of neural origin) and the brains of implanted rats. This work adds to the ongoing research on the polymers that are potentially suitable for neural implants because of their shape, flexibility, and elasticity [21].

In the case of NY, PCL, PDMS, and PEGDA, we observed poor growth and adhesion for both fibroblasts and neurons. It appears that the surfaces of these polymers have topographical and/or chemical properties which hinder cell adhesion and/or proliferation. For example, the polymer surface could be too smooth or have an irregular structure. Perhaps these polymers have low hydrophilicity and wettability, and hydrophobic surfaces are often unfavorable for cell adhesion [22]. The lack of cell growth limits the integration of the implant into the brain tissue, which reduces its effectiveness.

Among the structural factors, a polymer or products of its degradation could be toxic to cells [23]. Thus, we performed a cytotoxic test in the conditioned medium with polymers extracts. These experiments have shown that only PEGDA exhibited significant toxicity (Figure 4).

However, a polymer that does not support the growth of several cell types, especially fibroblasts, minimizes the likelihood of scar tissue formation and the development of an inflammatory response around the implant. The absence of a fibrotic or glial scar near the electrodes of the neural interface improves signal quality and reduces interference, which increases the accuracy of recordings and the efficacy of neurostimulation.

The polymers PET, PLA, and TPU exhibited properties that favored fibroblast growth and had a negative effect on the growth and/or adhesion of neurons. These compounds showed no signs of toxicity, which indicated that the exact structural and/or chemical characteristics of the substrate surface are not preferred for neuronal growth.

Several studies have shown the influence of chemical groups on the substrate surface on cell growth. In particular, certain chemical groups (e.g., the arginine–glycine–aspartate sequence) stimulate the growth of neurons [24]. There are other functional groups that ensure effective adhesion of cells to the surface, including fibroblasts [25]. Such a polymer could be suboptimal in connection with the formation of fibrosis at the implantation sites. Additionally, there is also a risk of neuroinflammation when such polymers are in close proximity to the nerve tissue, as fibroblasts can activate an immune response. The ability to direct the growth of nerve cells in certain directions due to the rejection effect can be used to gain certain advantages. However, such properties are better utilized in those polymers that do not support the growth of both neurons and fibroblasts.

The PP, PI, and PET-G scaffolds demonstrated that both neurons and fibroblasts grew. The effectiveness of this growth was variable, ranging from moderate for PP and PET-G to the best for PI. On the PI surface, the neurons adhered and grew almost as well as on the standard culture plastic, and the fibroblasts less well. Earlier, PI was also suggested as one of the most preferred materials for neural interfaces [26,27].

It is likely that the surface of these polymers had a surface that was optimal for the different cell types. For instance, the stiffness of the polymer could also have an influence, since cells from different organs could sense this substrate characteristic [28,29]. Neurons often prefer softer materials, while fibroblasts could also grow on stiff materials [30]. Moreover, it is well known that neurons grow better on scaffolds that have microchannel or anisotropic patterns in their structure, responding by aligning and elongating in the direction of these grooves [31,32].

A good compatibility of the surface of the implant with the different cells can facilitate its integration into the neuronal tissue [33]. This can improve the effectiveness of the recording and stimulation [34]. However, the growth of fibroblasts can lead to the formation of scar tissue around the implant, which can limit its functionality and increase the risk of rejection [35]. In this sense, PI is more interesting as it is more favorable to neurons than fibroblasts. However, fibroblasts play an important role in the healing after damage, and their presence around the implant can contribute to faster and more effective tissue repair in the absence of fibrosis and scar formation [36]. The growth of neurons on the implant surface can be viewed rather positively, as it can contribute to neuroplasticity, sprouting of neurites and axons along the surface of the implant, and thus to better functional integration and signal conduction.

Following the tests in culture, the compatibility of the investigated polymers with neural tissue was further tested by implantating them in the rat brain. After 4 post-implantation weeks, no severe FBR to NY, PCL, PDMS, PET, PET-G, PLA, PI, PP, and TPU was observed. On the contrary, PEGDA exhibited strikingly worse results with typical signs of inflammatory granuloma formation and the presence of foreign body cells, fibrotic capsule formation, and material migration. These reactions could not only compromise implant functionality but also cause damage to the brain.

It is worth noting that we did not observe a pronounced increase in fibrosis, even for the samples that could promote fibroblast growth. Moreover, PET, PDMS, and TPU with an unattractive surface interface (Figure 1) for both cell types even showed a slight decrease in negative effects in the brain compared to sham-operated animals. Given that these materials had low toxicity, the absence of cell growth on their surface could be an additional advantage for the use of these materials in neural interfaces.

Additionally, when designing an experiment to test the biocompatibility of neuroimplants, it is important to consider possible confounders. For instance, several anesthetics (for example, volatile anesthetics like isoflurane and sevoflurane) used in rodent experimental models have a neuroprotective effect confirmed by numerous studies [37,38]. Although chloral hydrate can exert a neuroprotective effect when animals are preconditioned with it [39,40], as an anesthetic agent, it does not affect this [38,41]. In an effort to exclude confounding factors as much as possible, we chose chloral hydrate as the anesthetic agent in this study. However, depending on the intended purpose, other options could be considered in future studies.

We also highlight the importance of long-term biocompatibility testing, especially for the materials that are prone to degradation. From the point of view of experimental in vivo modeling of the FBR, the effects at implantation periods longer than 7–14 days are often considered as chronic [42,43,44]. At implantation periods of 2–4 weeks or more, signs of tissue remodeling already appear, and the initial stages of fibrous/glial scar formation are present [9,45]. However, as was also shown in this study, in the case of PEGDA, degradation of the material can lead to migration of its particles. And if the observation period is not long enough, signs of FBR may not yet appear, particularly the ones caused by an uncontrolled migration of polymers.

This study showed that PI appeared to be the most promising material for further analysis and implementation in implants for neural interfaces, as it is the most biocompatible and neurotrophic. PLA, PDMS, and TPU could be practical, as well, as they do not appear to stimulate neuroregeneration and are mostly inert to brain tissue. PEGDA appears to be the material with multiple negative effects, so it does not appear of any use for further development.

## 4. Materials and Methods

### 4.1. The Preparation of Polymer Scaffolds

The following materials were used in the study: NY, PCL, PEGDA, PDMS, PET, PLA, TPU, PP, PET-G, and PI. From these polymers, two types of samples were prepared: disks with a diameter of 15 mm and 1 mm thickness for the in vitro experiments and cylinders with a diameter of 0.9 mm and 3 mm length for the in vivo experiments.

NY (Ultimaker, Utrecht, The Netherlands), PET (Innofil3D, Emmen, The Netherlands), PET-G (ABS Maker, Khimki, Russia), PLA (Innofil3D, Emmen, The Netherlands), PCL (ESUN, Shenzhen, China), PP (Ultimaker, Utrecht, The Netherlands), and TPU (Eryone, Shenzhen, China) scaffolds for in vitro experiments were produced using 3D printing (Ultimaker 2+, Ultimaker, Utrecht, The Netherlands) from the filament with the following parameters. The filament was pre-dried for 24 h at 50 °C (TPU, PP, NY, PLA, PET, and PET-G) or 30 °C (PCL). The nozzle temperature was 200 °C (PCL), 210 °C (TPU, PLA, PET, and PET-G), 230 °C (PP), or 260 °C (NY). The print bed temperature was 50 °C (PCL), 60 °C (TPU, PLA, PET, PET-G, and NY), or 80 °C (PP). 3D printing was performed in the open box with the fan on (PCL, PLA) or in the enclosed box with the fan off (TPU, PP, NY, PET, and PET-G). The printing speed was 50 mm/s (TPU, PLA, PET, and PET-G) or 60 mm/s (PP—first two layers with 5 mm/s speed, NY, PCL). During the printing, the thickness of each layer of all polymers was 0.1 mm, and the wall thickness was 0.1–0.3 mm.

The PDMS samples were prepared by casting SMARTSIL ST-501 (BMP Technology, Moscow, Russia) (the ratio of A:B components was 1:1). The PI samples were prepared by casting 13% polyimide varnish (ESTROKOM, Moscow, Russia) into a mold to obtain a disk thickness of 6–7 mm. After drying at 60 °C for 24 h, disks with a diameter of 16 mm were cut with a CO_2_ laser (processing speed of 10.5 mm/s and power of 12%). The disks were placed between two glasses and fired at 260 °C for 1 h at a heating rate of 100 °C/h. The PEGDA samples were prepared by casting a solution of 20% PEGDA Mn = 700 (Sigma Aldrich, St. Louis, MO, USA) in ultra-pure water (produced by UPD-I-10T device, Ulupure, Chengdu, China) with an addition of 1% API-TPO photoinitiator (CPS, Franklin, KY, USA) into the mold. The solution was poured into a silicone mold and polymerized under a UV lamp for 2 min. The samples were washed successively with isopropyl alcohol and distilled water to remove residual photoinitiator.

NY, PET, PET-G, PLA, PCL, PP, and TPU cylindrical non-functional implants for in vivo experiments were produced by thermal extrusion of the polymer. PEGDA, PDMS and PI samples were cast into a mold. The filament of the extrusion 3D printer was melted on a hotplate at 220 °C and given a cubic shape. Then it was cooled at room temperature until completely solidified. The sample was placed in a vice and extracted with a sharpened 18G medical needle. The resulting cylinder of the target material was shortened to obtain a sample length of 3 mm.

The PDMS sample was prepared by casting SMARTSIL ST-501 (1:1) into a Petri dish. To accelerate crosslinking, PDMS was heated on a hotplate at 80 °C for 2 h. The samples were extracted with a 16G needle. Then they were further processed with an 18G needle to a diameter of 0.9 mm. The PI sample was prepared by casting 13% polyimide varnish into a Petri dish to be dried in an oven at 60 °C for 12 h. The samples were extracted with a 16G needle and fired in an oven at 260 °C for 1 h before final processing with an 18G needle. The PEGDA samples were prepared by casting a solution of 20% PEGDA Mn = 700 in ultra-pure water with an addition of 1% API-TPO photoinitiator into the mold. The solution was polymerized under a UV lamp for 2 min. The samples were washed successively with isopropyl alcohol and distilled water to remove residual photoinitiator. Samples were extracted with a 16G needle. The PEGDA samples lose water in the air and decrease to the target diameter of 0.9 mm.

### 4.2. Surface Characterization Using SEM

For the scanning electron microscopy (SEM) study, a layer of chromium was deposited on the samples with a sputter coater (QT-150T ES, Quorum Technologies, Laughton, East Sussex, UK). SEM images were obtained using an Amber GMH (Tescan, Brno, Czech Republic) microscope operated at an accelerating voltage of 2 kV using a secondary electron detector. These experiments were performed using the equipment of the JRC PMR IGIC RAS.

### 4.3. Cell Cultures and Biocompatibility Assessment

To examine the biocompatibility of the polymer disks, we performed experiments on NRK-49F and PC-12. The cells were cultured in DMEM/F12 supplemented with glutamine (PanEco, Moscow, Russia) and 5 or 10% fetal bovine serum (FBS) (HiMedia, Maharashtra, India) in a CO_2_ incubator (Eppendorf, Hamburg, Germany) at 37 °C and 5% CO_2_. Prior to the experiment, we performed sterilization of the polymer disks using a UV lamp with 185 nm and 254 nm emission. Both sides of the scaffolds were exposed to UV. Then, the polymers were placed in the wells of 6-well plates and were covered with a suspension of appropriate cells. After 48 h of cultivation, the staining with calcein-AM (Invitrogen, Carlsbad, CA, USA) was performed to label the living cells on the substrate. Propidium iodide staining (Lumiprobe, Moscow, Russia) was used to detect the dead cells. After staining, the polymers with the covering cells were transferred to glass-bottomed dishes and examined using an LSM900 confocal microscope (Carl Zeiss, Baden-Württemberg, Germany). Calcein-AM was visualized using blue laser excitation (488 nm), and fluorescence emission was analyzed at 505–530 nm. Propidium iodide accumulation was analyzed using 543 nm excitation, and emission was detected in the range of 560–590 nm. For each polymer, 30 fields of view were used in three independent series of experiments. Then, quantitative analysis of confocal images was performed using software implemented in Python version 3.9.

### 4.4. In Vitro Toxicity Assessment of Scaffolds

To determine the potential toxic effects of soluble substrate components, conditioned media were prepared by incubating the substrate for 48 h in the same growth medium and under the same conditions as for the cell growth assay on the substrate. For toxicity assays, PC-12 or NRK-49F cells were cultured in a 96-well plate. Then, the standard growth medium was replaced with the substrate-conditioned medium composed of the appropriate polymer and cells. The medium was incubated for 48 h. Then, a standard MTT test was performed. MTT solution (1 mg/mL) was added to the cells for 1 h, then discharged, and the cells were lysed with dimethyl sulfoxide (DMSO) (PanEco, Moscow, Russia). Absorbance was measured at 540 nm using a Zenyth 3100 plate multimode detector (Anthos Labtec, Wals, Austria). Wells containing cells incubated with H_2_O for 48 h were used as negative controls.

### 4.5. Animal Experiments

The in vivo experiments were performed on male (*n* = 44) young outbred Wistar rats (3–4-month-old, 300–400 g). The animal protocol was examined and approved by the animal ethics committee of A.N. Belozersky Institute of Physico-Chemical Biology Lomonosov Moscow State University, Protocol № 010-5/05/2024. All procedures were conducted in accordance with the “Animal Research: Reporting of In Vivo Experiments” (ARRIVE) guidelines. The animals had unlimited access to food and water and were kept in cages in a temperature-controlled environment (20 ± 1 °C) under a 12/12 h light/dark cycle.

Animals were anesthetized using chloral hydrate (300 mg per kg of body weight). After the animal reached a sufficient depth of anesthesia and did not exhibit reflexes, an incision was carefully made over the skull midline. The remaining epidermis and connective tissues were removed with hydrogen peroxide. A craniotomy was performed through careful drilling using a dental drill. The insertion of phantom implants into the brain was performed using an 18G needle with a tip cut at a 90-degree angle. The implant was inserted stereotactically following the coordinates AP-3.6, ML-2.5, and DV-8.3. To ensure precise implantation, a custom fixture was designed for the stereotaxic frame, enabling the needle to reach the desired depth in the brain. During the process of withdrawal, the needle was carefully extracted in a controlled manner, leaving the cylindrical implant in a certain location in the brain. This was achieved by securing the implant at the back of the needle with a cylindrical rod (0.9 mm in diameter), which remained stationary while the needle was removed.

Four weeks after the surgery, the animals were deeply anesthetized with xylazine (5 mg/kg IP) and Zoletil 100 (Virbac, Carros, France) (40 mg/kg IP). After the anesthesia administration, sequential transcardiac perfusion with 100 mL of saline and 100 mL of 4% neutral buffered formaldehyde (NBF) (Biovitrum, Moscow, Russia) was performed. The completion of transcardiac perfusion was assured by the assessment of muscle rigidity and inner organs paleness. Then, the brain was carefully taken out of the skull and immersed in NBF for 24–48 h.

### 4.6. Histological Evaluation

2 mm-thick brain sections containing the implantation site were trimmed in the coronal plane, washed for 20 min in tap water, and processed to paraffin wax in an automatic tissue processor Leica ASP6025S (Leica Biosystems, Nussloch, Germany) according to standard protocol [46]. Afterwards, samples were embedded into paraffin wax with higher melting point Histomix (Biovirtrum, Moscow, Russia) using Leica HistoCore Arcadia embedding station (Leica Biosystems, Nussloch, Germany). Most of the implants were removed either at the trimming stage or during microtomy due to the highly pronounced difference in stiffness between the implant material and brain tissue. However, it was feasible to obtain thin sections of most brain samples containing implants composed of PEGDA and polyimide.

Samples were sectioned on a Thermo Fisher HM 340E rotary microtome (ThermoFisher Scientific, Waltham, MA, USA) in non-freezing conditions. Samples were initially trimmed until the implantation zone was visible in the sections. Next, thin sections were prepared with a thickness of 2–3 µm for hematoxylin and eosin staining and 5–6 µm for the other stains. Before staining, sections were vertically dried for at least 1 h at +37 °C.

In order to assess the general condition of the implantation site, the sections were stained with hematoxylin and eosin according to the conventional protocol. For visualization of neurons and glia, the sections were stained with cresyl violet solution. For the assessment of fibrotic scar formation and visualization of hemosiderin, Mallory’s trichrome stain kit (Biovitrum, Moscow, Russia) and Perls’ Prussian blue staining kit (Biovitrum, Moscow, Russia) were used according to the manufacturer’s instructions. The brain tissue reaction to implants composed of different materials was evaluated semi-quantitatively according to the criteria presented in Table 1. Visual examples of scores are provided in the Supplementary Materials (Table S1).

For histological evaluation, 44 samples from 11 groups were analyzed: (1) sham operated (*n* = 4); (2) Ny (*n* = 6); (3) PCL (*n* = 3); (4) PDMS (*n* = 4); (5) PEGDA (*n* = 4); (6) PET (*n* = 3); (7) PET-G (*n* = 4); (8) PLA (*n* = 5); (9) Polyimide (*n* = 3); (10) PP (*n* = 4); (11) TPU (*n* = 4). Samples were excluded from the analysis if severe ventriculomegaly was observed. Severe ventriculomegaly cases were sporadic (2 cases in total) and observed in different groups (PLA and PP, 1 case per group), while the other animals in the same groups did not exhibit it. The development of ventriculomegaly likely occurred due to an incidental displacement during surgical implantation. Due to the alterations caused by severe ventriculomegaly, these samples were excluded from the analysis.

### 4.7. Statistics

The statistical data analysis and data visualization were performed using the GraphPad Prism 9 software (GraphPad Software Inc., Boston, MA, USA). The sample data were tested for normality using the Shapiro–Wilk test. For cell culture experiments, ANOVA with Tukey’s multiple comparisons test was used in the cases of parametric variables, and Kruskal–Wallis test with Dunn’s multiple comparisons was used in the cases of nonparametric variables. For histological data, statistical significance was evaluated using two-way ANOVA with Dunnett’s multiple comparisons test. *p* < 0.05 was admitted statistically significant. The values within the group were expressed as mean ± SEM.

## Figures and Tables

**Table 1:** Histological score.

Feature	Score	Description
Cell death	0	None, or only in the area of injection in the cortex
	1	Degraded neurons in the peri-implant area
	2	Massive degeneration of neurons at the peri-implant area with edema of neuropil
	3	Extensive degeneration of neurons and neuropil deterioration with active immune response
Infiltration rate	0	None
	1	Mild: presence of activated glial cells and rare macrophages
	2	Moderate: Focal dense immune infiltrate containing glial cells, macrophages, and lymphocytes
	3	Severe: Granuloma formation with multinucleated giant cells
Fibrosis	0	None
	1	Sparse collagen fibers are non-uniformly spread around the implant
	2	Uniform layer of collagen fibers all around the implant
	3	Extensive fibrotic scar
Ventricles enlargement	0	None
	1	Unilateral ventricle enlargement
	2	Bilateral ventricle enlargement

## Data Availability

Data is contained within the article or Supplementary Materials.

## Supplementary Materials

The following supporting information can be downloaded at: https://www.mdpi.com/article/10.3390/bios15090599/s1, Table S1: Histological score.

## Abbreviations

The following abbreviations are used in this manuscript:

DMSO	Dimethyl sulfoxide
FBR	Foreign body reaction
FBS	Fetal bovine serum
NBF	Neutral buffered formaldehyde
NY	Nylon 618
PCL	Polycaprolactone
PDMS	Polydimethylsiloxane
PEGDA	Polyethylene glycol diacrylate
PET	Polyethylene terephthalate
PET-G	Polyethylene terephthalate glycol
PI	Polyimide
PLA	Polylactide
PP	Polypropylene
SEM	Scanning electron microscopy
TPU	Thermoplastic polyurethane

## References

[1] Shen K., Chen O., Edmunds J.L., Piech D.K., Maharbiz M.M. (2023). Translational Opportunities and Challenges of Invasive Electrodes for Neural Interfaces. Nat. Biomed. Eng..

[2] Vallejo-Giraldo C., Genta M., Goding J., Green R. (2021). Biomimetic Approaches towards Device-Tissue Integration. Handbook of Neuroengineering.

[3] Valeriani D., Santoro F., Ienca M. (2022). The Present and Future of Neural Interfaces. Front. Neurorobotics.

[4] Larson C.E., Meng E. (2020). A Review for the Peripheral Nerve Interface Designer. J. Neurosci. Methods.

[5] Gori M., Vadalà G., Giannitelli S.M., Denaro V., Di Pino G. (2021). Biomedical and Tissue Engineering Strategies to Control Foreign Body Reaction to Invasive Neural Electrodes. Front. Bioeng. Biotechnol..

[6] Williams D.F. (2008). On the Mechanisms of Biocompatibility. Biomaterials.

[7] Budday S., Nay R., de Rooij R., Steinmann P., Wyrobek T., Ovaert T.C., Kuhl E. (2015). Mechanical Properties of Gray and White Matter Brain Tissue by Indentation. J. Mech. Behav. Biomed. Mater..

[8] Weltman A., Yoo J., Meng E. (2016). Flexible, Penetrating Brain Probes Enabled by Advances in Polymer Microfabrication. Micromachines.

[9] Carnicer-Lombarte A., Chen S.-T., Malliaras G.G., Barone D.G. (2021). Foreign Body Reaction to Implanted Biomaterials and Its Impact in Nerve Neuroprosthetics. Front. Bioeng. Biotechnol..

[10] Lotti F., Ranieri F., Vadalà G., Zollo L., Di Pino G. (2017). Invasive Intraneural Interfaces: Foreign Body Reaction Issues. Front. Neurosci..

[11] Gou S., Yang S., Cheng Y., Yang S., Liu H., Li P., Du Z. (2024). Applications of 2D Nanomaterials in Neural Interface. Int. J. Mol. Sci..

[12] Klopfleisch R., Jung F. (2017). The Pathology of the Foreign Body Reaction against Biomaterials. J. Biomed. Mater. Res. Part A.

[13] Gulino M., Kim D., Pané S., Santos S.D., Pêgo A.P. (2019). Tissue Response to Neural Implants: The Use of Model Systems toward New Design Solutions of Implantable Microelectrodes. Front. Neurosci..

[14] Prodanov D., Delbeke J. (2016). Mechanical and Biological Interactions of Implants with the Brain and Their Impact on Implant Design. Front. Neurosci..

[15] Hatsopoulos N.G., Donoghue J.P. (2009). The Science of Neural Interface Systems. Annu. Rev. Neurosci..

[16] Sung C., Jeon W., Nam K.S., Kim Y., Butt H., Park S. (2020). Multimaterial and Multifunctional Neural Interfaces: From Surface-Type and Implantable Electrodes to Fiber-Based Devices. J. Mater. Chem. B.

[17] Ferguson M., Sharma D., Ross D., Zhao F. (2019). A Critical Review of Microelectrode Arrays and Strategies for Improving Neural Interfaces. Adv. Healthc. Mater..

[18] Wunderlich H., Kozielski K.L. (2021). Next Generation Material Interfaces for Neural Engineering. Curr. Opin. Biotechnol..

[19] Hassler C., Boretius T., Stieglitz T. (2011). Polymers for Neural Implants. J. Polym. Sci. Part B Polym. Phys..

[20] Green R.A., Lovell N.H., Wallace G.G., Poole-Warren L.A. (2008). Conducting Polymers for Neural Interfaces: Challenges in Developing an Effective Long-Term Implant. Biomaterials.

[21] Lecomte A., Descamps E., Bergaud C. (2018). A Review on Mechanical Considerations for Chronically-Implanted Neural Probes. J. Neural Eng..

[22] Bacakova L., Filova E., Parizek M., Ruml T., Svorcik V. (2011). Modulation of Cell Adhesion, Proliferation and Differentiation on Materials Designed for Body Implants. Biotechnol. Adv..

[23] Fischer D., Li Y., Ahlemeyer B., Krieglstein J., Kissel T. (2003). In Vitro Cytotoxicity Testing of Polycations: Influence of Polymer Structure on Cell Viability and Hemolysis. Biomaterials.

[24] Galindo J.M., San-Millán M.I., Castillo-Sarmiento C.A., Ballesteros-Yáñez I., Vázquez E., Merino S., Herrero M.A. (2024). Optimization of 3D Synthetic Scaffolds for Neuronal Tissue Engineering Applications. Chemistry.

[25] Aye S.-S.S., Li R., Boyd-Moss M., Long B., Pavuluri S., Bruggeman K., Wang Y., Barrow C.R., Nisbet D.R., Williams R.J. (2018). Scaffolds Formed via the Non-Equilibrium Supramolecular Assembly of the Synergistic ECM Peptides RGD and PHSRN Demonstrate Improved Cell Attachment in 3D. Polymers.

[26] Myllymaa S., Myllymaa K., Korhonen H., Djupsund K., Tanila H., Lappalainen R. (2008). Development of Flexible Thin Film Microelectrode Arrays for Neural Recordings. 14th Nordic-Baltic Conference on Biomedical Engineering and Medical Physics, Riga, Latvia, 16–20 June 2008.

[27] Kumar K., Deshpande K., Kalur N., Chauhan G., Chugh D., Ganesh S., Ramakrishnan A. (2023). Polyimide-Based Flexible Multi-Electrode Arrays: Synthesis, Microfabrication, and In-Vivo Validation. bioRxiv.

[28] Deroanne C.F., Lapiere C.M., Nusgens B.V. (2001). In Vitro Tubulogenesis of Endothelial Cells by Relaxation of the Coupling Extracellular Matrix-Cytoskeleton. Cardiovasc. Res..

[29] Engler A., Bacakova L., Newman C., Hategan A., Griffin M., Discher D. (2004). Substrate Compliance versus Ligand Density in Cell on Gel Responses. Biophys. J..

[30] Discher D.E., Janmey P., Wang Y.-L. (2005). Tissue Cells Feel and Respond to the Stiffness of Their Substrate. Science.

[31] Shahriari D., Loke G., Tafel I., Park S., Chiang P.-H., Fink Y., Anikeeva P. (2019). Scalable Fabrication of Porous Microchannel Nerve Guidance Scaffolds with Complex Geometries. Adv. Mater..

[32] Marcus M., Baranes K., Park M., Choi I.S., Kang K., Shefi O. (2017). Interactions of Neurons with Physical Environments. Adv. Healthc. Mater..

[33] Boulingre M., Portillo-Lara R., Green R.A. (2023). Biohybrid Neural Interfaces: Improving the Biological Integration of Neural Implants. Chem. Commun..

[34] Kumosa L.S. (2023). Commonly Overlooked Factors in Biocompatibility Studies of Neural Implants. Adv. Sci..

[35] Wurth S., Capogrosso M., Raspopovic S., Gandar J., Federici G., Kinany N., Cutrone A., Piersigilli A., Pavlova N., Guiet R. (2017). Long-Term Usability and Bio-Integration of Polyimide-Based Intra-Neural Stimulating Electrodes. Biomaterials.

[36] Dorrier C.E., Jones H.E., Pintarić L., Siegenthaler J.A., Daneman R. (2022). Emerging Roles for CNS Fibroblasts in Health, Injury and Disease. Nat. Rev. Neurosci..

[37] Scheid S., Goebel U., Ulbrich F. (2023). Neuroprotection Is in the Air—Inhaled Gases on Their Way to the Neurons. Cells.

[38] Archer D.P., McCann S.K., Walker A.M., Premji Z.A., Rogan K.J., Hutton M.J.H., Gray L.J. (2018). Neuroprotection by Anaesthetics in Rodent Models of Traumatic Brain Injury: A Systematic Review and Network Meta-Analysis. Br. J. Anaesth..

[39] Silachev D.N., Usatikova E.A., Pevzner I.B., Zorova L.D., Babenko V.A., Gulyaev M.V., Pirogov Y.A., Plotnikov E.Y., Zorov D.B. (2017). Effect of Anesthetics on Efficiency of Remote Ischemic Preconditioning. Biochemistry.

[40] Liu J.-H., Feng D., Zhang Y.-F., Shang Y., Wu Y., Li X.-F., Pei L. (2015). Chloral Hydrate Preconditioning Protects against Ischemic Stroke via Upregulating Annexin A1. CNS Neurosci. Ther..

[41] Bocharnikov A.D., Boeva E.A., Milovanova M.A., Antonova V.V., Yakupova E.I., Grechko A.V. (2024). Nueroprotection by Anesthetics in Brain Injury Models. Gen. Reanimatol..

[42] Du Z.J., Kolarcik C.L., Kozai T.D.Y., Luebben S.D., Sapp S.A., Zheng X.S., Nabity J.A., Cui X.T. (2017). Ultrasoft Microwire Neural Electrodes Improve Chronic Tissue Integration. Acta Biomater..

[43] Azemi E., Gobbel G.T., Cui X.T. (2010). Seeding Neural Progenitor Cells on Silicon-Based Neural Probes. J. Neurosurg..

[44] Adewole D.O., Struzyna L.A., Burrell J.C., Harris J.P., Nemes A.D., Petrov D., Kraft R.H., Chen H.I., Serruya M.D., Wolf J.A. (2021). Development of Optically Controlled “Living Electrodes” with Long-Projecting Axon Tracts for a Synaptic Brain-Machine Interface. Sci. Adv..

[45] Gilmour A.D., Woolley A.J., Poole-Warren L.A., Thomson C.E., Green R.A. (2016). A Critical Review of Cell Culture Strategies for Modelling Intracortical Brain Implant Material Reactions. Biomaterials.

[46] Savchenko E.S., Pevzner I.B., Zorova L.D., Silachev D.N., Babenko V.A., Manskikh V.N., Gulyaev M.V., Pirogov Y.A., Plotnikov E.Y., Zorov D.B. (2016). Changes in the Number of Neurons, Astrocytes and Microglia in the Brain After Ischemic Stroke Assessed by Immunohistochemistry and Immunoblotting. Tsitologiia.

