# Supplementary material for: Foreign Body Reaction to Neural Implants: A Comparative Study of Polymer Toxicity and Tissue Response
Source: Biosensors (Basel). 2025 Sep 11;15(9):599. doi: 10.3390/bios15090599 (PMC12467080; doi:10.3390/bios15090599)
Supplement: Supplementary file 1 [file biosensors-15-00599-s001.zip › biosensors-3817528-supplementary.pdf]

Supplementary Materials

Table S1. Histological score.

**Cell death**		**Infiltration rate**		**Fibrosis**		**Ventricles enlargement**	
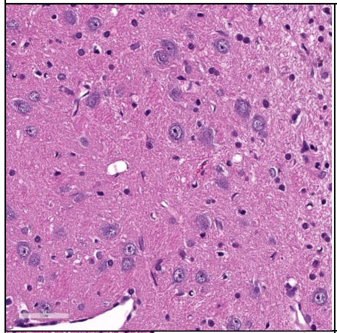	**Score 0**: None, or only at the area of injection in cortex	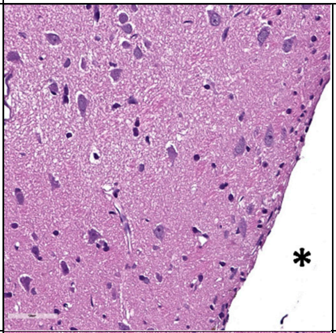	**Score 0**: None	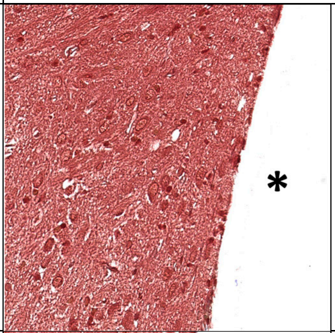	**Score 0**: None	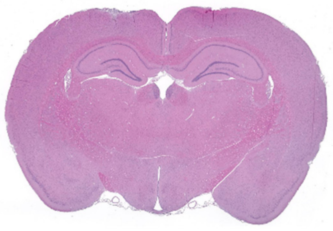	**Score 0**: None
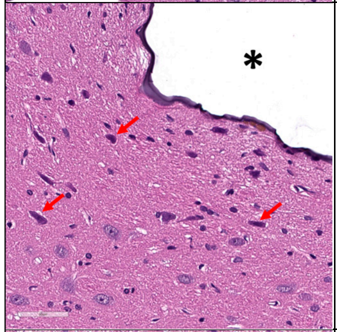	**Score 1**: Degenerated neurons at peri-implant area	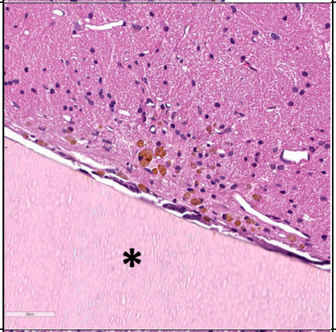	**Score 1**: Mild. Presence of activated glial cells and rare macrophages	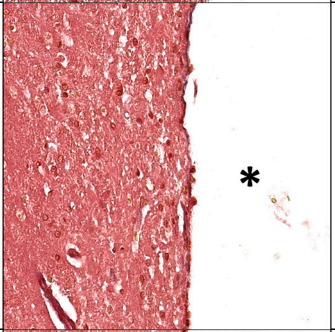	**Score 1**: Sparse collagen fibers non-uniformly spread around implant	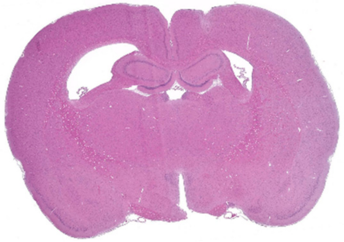	**Score 1**: Unilateral ventricle enlargement
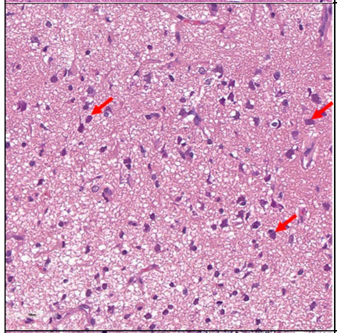	**Score 2**: Massive degeneration of neurons at the peri-implant area with edema of neuropil	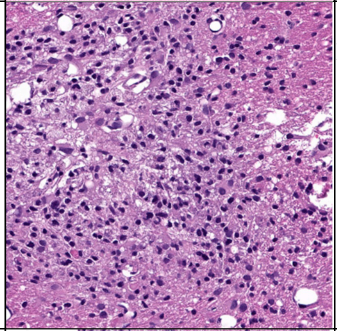	**Score 2**: Moderate. Focal dense immune infiltrate containing glial cells, macrophages and lymphocytes	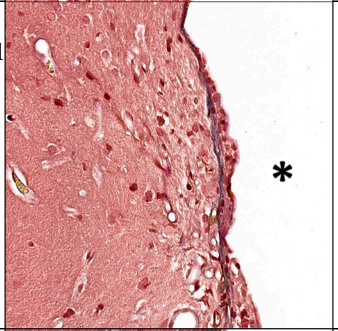	**Score 2**: Uniform layer of collagen fibers all around implant	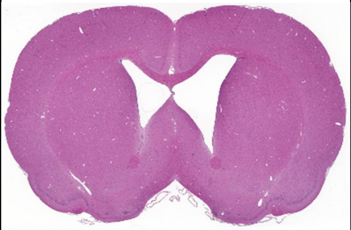	**Score 2**: Bilateral ventricle enlargement
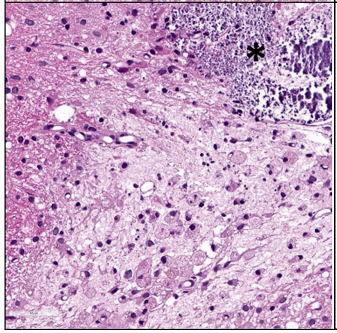	**Score 3**: Extensive degeneration of neurons and neuropil deterioration with active immune response	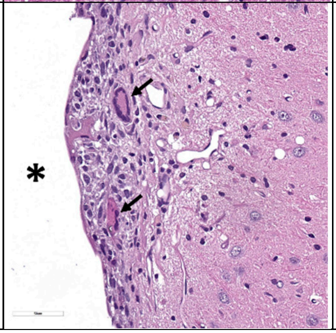	**Score 3**: Severe. Granuloma formation with multinucleated giant cells	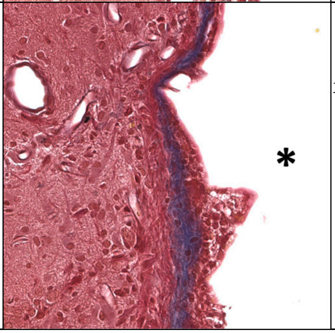	**Score 3**: Extensive fibrotic scar	* - implantation cavity<br>← - degenerated neurons<br>← - multinucleated giant cell<br><br>Staining:<br>Cell death, Infiltration, and Ventricles enlargement – H&E;<br>Fibrosis – Mallory's trichrome stain (collagen fibers stained blue).	
